# Supplementary material for: Using ethics of care as the theoretical lens to understand lived experiences of caregivers of older adults experiencing functional difficulties
Source: PLoS One. 2022 May 5;17(5):e0267658. doi: 10.1371/journal.pone.0267658 (PMC9071116; doi:10.1371/journal.pone.0267658)
Supplement: S1 File — (DOCX) [file pone.0267658.s001.docx]

Interview schedule

**Study 2: Exploring the lived experiences of caregivers who provide care and support to older people**

**Objective 1: Gain an in-depth understanding of the nature, extent, and motivation of care and support adult children provide for older relative.**

1. Please can you tell me how you come to be the caregiver for the cared-for?

(***Prompt***: Can you tell me about where you were before? What you did before taking the role as a caregiver to the cared-for? How did you arrive at your decision as a caregiver to the cared-for?

1. Can you describe to me the nature of care and support you provide to the cared-for?

(***Prompt***: What kind of care and support do you provide? How do you provide the care and support? To what extent do you provide the care and support?

1. Can you explain to me your reasons for providing care and support to the cared-for?

**Prompt:** What is your motivations for providing the care and support to the older relative? Why did you arrive at your decision as a caregiver to the cared-for?

**Objective 2: Examine the context of support provision and factors affecting its provision, including demographic characteristics and socioeconomic status**

1. Can you tell me what affect the nature of care and support you provide for the cared-for?

**Prompt:** What makes it easy? What makes it difficult? Family demands, socioeconomic status, intensity of care needs of the cared-for etc

**Objective 3: Understand sibling relations, primary caregiver’s perception of siblings’ relations and the sharing of the responsibilities for parental care.**

1. Can you tell me about your siblings’ participation level in the care and support you provide to the cared-for?

***Prompt*:** How do your siblings assist in parental care? Can you explain to me the reasons for your siblings’ participation level in caregiving?

1. Tell me, how do you perceive the nature of your sibling participation level regarding parental caregiving duties?
2. Can you tell me if you will ever ask your siblings to share in the caregiving responsibilities to the cared-for? (if sibling relation or participation is absence or minimal)

**Objective 4: Understand the experiences of caregivers regarding caregiving duties.**

1. How will you describe yourself as a caregiver to the cared-for?

**Prompt:** how do you see yourself?

1. Could you please describe to me your best experience concerning the care you provide?

**Prompt:**

1. Could you please describe to me your worst experience concerning the care you provide?

**Prompt:** Could you explain to me the cost you have incurred because of caregiving? (lost of job, loss of social network)

1. Can you describe to me how caregiving to the cared-for has affected your relationship with other people?

**Prompt** partner, family, friends, work colleagues

**Objectives 5: Explore adult children’s coping strategies and resilience, as well as the stresses and strains in providing support for their older people.**

1. Please can you explain to me how you cope with caregiving stresses and strains?

**Prompt:** how do you deal with the challenges that you encounter because of caregiving duties? Describe to me what has been helping you to continue in the support and care to the cared-for (prayer, help from others, ability to blend care work with work,)

1. What is your experience with support you receive from others (except siblings) due to the caregiving to the cared-for (if any)?

**Prompt:** government/non-government, friends,

**Objectives 6: Understand adult children’s willingness to continue providing care and support for older people.**

1. Can you please tell me if you will be able to continue providing care and support the cared-for in the future?

Prompt:
